# The AURORA pilot study for molecular screening of patients with advanced breast cancer–a study of the breast international group

**DOI:** 10.1038/s41523-017-0026-6

**Published:** 2017-06-29

**Authors:** Marion Maetens, David Brown, Alexandre Irrthum, Philippe Aftimos, Giuseppe Viale, Sibylle Loibl, Jean-François Laes, Peter J. Campbell, Alastair Thompson, Javier Cortes, Sabine Seiler, Sara Vinnicombe, Mafalda Oliveira, Françoise Rothé, Yacine Bareche, Debora Fumagalli, Dimitrios Zardavas, Christine Desmedt, Martine Piccart, Sherene Loi, Christos Sotiriou

**Affiliations:** 1 J.-C. Heuson Breast Cancer Translational Research Laboratory, Institut Jules Bordet, Université Libre de Bruxelles, Brussels, Belgium; 2 Breast International Group, Brussels, Belgium; 3 Department of Medical Oncology, Institut Jules Bordet, Université Libre de Bruxelles, Brussels, Belgium; 4 European Institute of Oncology, University of Milan, Milan, Italy; 5 Sana Klinikum, Offenbach, Germany and German Breast Group, Neu-Isenburg, Germany; 6 OncoDNA, Gosselies, Belgium; 7 Cancer Genome Project, Wellcome Trust Sanger Institute, Hinxton, UK; 8 Department of Breast Surgical Oncology, University of Texas MD Anderson Cancer Center, Houston, USA; 9 Dundee Cancer Centre, University of Dundee, Dundee, UK; 10 Ramon y Cajal University Hospital, Madrid, Spain; 11 Vall d´Hebron Institute of Oncology, Barcelona, Spain; 12 Division of Clinical Medicine and Research, Peter MacCallum Cancer Centre, Melbourne, Victoria Australia

## Abstract

Several studies have demonstrated the feasibility of molecular screening of tumour samples for matching patients with cancer to targeted therapies. However, most of them have been carried out at institutional or national level. Herein, we report on the pilot phase of AURORA (NCT02102165), a European multinational collaborative molecular screening initiative for advanced breast cancer patients. Forty-one patients were prospectively enroled at four participating centres across Europe. Metastatic tumours were biopsied and profiled using an Ion Torrent sequencing platform at a central facility. Sequencing results were obtained for 63% of the patients in real-time with variable turnaround time stemming from delays between patient consent and biopsy. At least one clinically actionable mutation was identified in 73% of patients. We used the Illumina sequencing technology for orthogonal validation and achieved an average of 66% concordance of substitution calls per patient. Additionally, copy number aberrations inferred from the Ion Torrent sequencing were compared to single nucleotide polymorphism arrays and found to be 59% concordant on average. Although this study demonstrates that powerful next generation genomic techniques are logistically ready for international molecular screening programs in routine clinical settings, technical challenges remain to be addressed in order to ensure the accuracy and clinical utility of the genomic data.

## Molecular screening: Multinational testing doable, but technical challenges remain

A pilot study demonstrated that a large-scale, international screening programme for women with metastatic breast cancer is feasible. The study, coordinated by the Institut Jules Bordet and the Breast International Group, aimed to determine whether biopsies and blood could be collected from women with metastatic breast cancer across Europe and sent to a central laboratory for targeted gene sequencing. Genetic information was successfully obtained for 26 of the 41 participants, 19 of whom had mutations that could be targeted with a known drug, potentially influencing treatment decision-making. They concluded that genomic testing is logistically ready for international molecular screening in routine clinical settings laying the groundwork for the parent European AURORA molecular screening programme which aims at recruiting 1300 metastatic breast cancer patients. However, technical challenges remain to be addressed to ensure the accuracy and robustness across different sequencing platforms.

## Introduction

Several efforts have advanced our understanding of the alterations characterizing cancer genomes.^1, 2^ Coupled to recent successes of targeted therapies in patients with molecularly profiled tumours^3, 4^ and the decreasing costs of massively parallel sequencing, this has motivated several studies, albeit of limited size, to investigate the implementation of personalised molecular screening in the clinical settings.^5–7^

Most of these studies were focused on primary tumours and despite growing evidence that distant metastases may harbour additional molecular alterations absent from their matched primaries,^8–12^ genomic information about metastatic disease remains limited. Even though the clinical relevance of many of these alterations remains to be established, it is increasingly recognised that molecular profiling of advanced disease could help elucidate the biological underpinnings of phenomena such as distant recurrence and the emergence of de novo resistance to therapy.^13^ Lastly, in order to find applications in routine clinical practice, it is essential to assess the reliability and robustness of the chosen sequencing platform using orthogonal sequencing strategies.^14, 15^

The Breast International Group launched AURORA–Aiming to Understand the Molecular Aberrations in Metastatic Breast Cancer, a pan-European molecular screening programme whose main goal is to deepen our knowledge of the genomic landscape of advanced breast cancer.^16^ Herein, we report on the pilot phase of this study whereby the primary objective was to investigate the feasibility with four European recruitment sites and central pathological and sequencing facilities. Secondary aims were to assess the concordance of somatic mutations between two targeted next generation sequencing (NGS) platforms and of somatic copy number aberrations (CNA) obtained from NGS and single nucleotide polymorphism (SNP) arrays.

## Results

### Patient recruitment and logistics

**Fig. 1:**
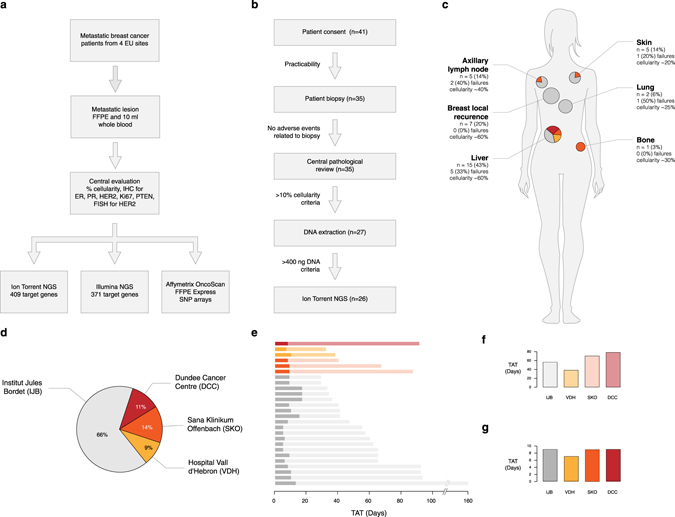
Logistics and feasibility of the study. **a** logistic workflow of the study, **b** inclusion criteria and number of patients with successful results, **c** anatomical distribution of biopsied lesions with breakdown by recruiting centre, **d** global distribution of patients by recruiting centre, **e** turnaround time for each patient with breakdown by recruiting centre, **f** median global turnaround time by recruiting centre, and **g** median delivery turnaround time by recruiting centre. In **a**, the Illumina sequencing and Affymetrix OncoScan SNP arrays were done as batch processes. 19 patients were sequenced by Illumina targeted NGS and 18 patients were genotyped using the Affymetrix SNP arrays. However, these are not fully overlapping subsets and only 14 (54%) patients had the full set of all three data types. In **a** and **b**, the Ion Torrent sequencing results were obtained in real-time and in (**e**), the darker shades indicate delivery turnaround time (TAT) whilst the lighter shades indicate global turnaround time. The colour codes for the recruiting centres in **c** to **g** are indicated in (**d**)

A total of 41 patients provided informed consent and were enroled in this pilot study between February 2013 and September 2014. Fig. 1a and b illustrates the study design and Table 1 summarises the clinical and pathological characteristics of the patients. Formalin fixed paraffin embedded (FFPE) biopsies of metastatic lesions and whole blood were prospectively collected from 35 (85%) of them. 20 (57%) patients had ER + metastatic disease, 7 (20%) were HER2 + and 8 (23%) were triple negative breast cancer. Fig. 1c and d represent the distribution of the different anatomical sites and the breakdown of patients’ recruitment by participating centre. Following central pathological review, a tumour content below 10% was recorded for 8 (23%) patients whilst the median cellularity was 50% (range 10–85). One additional sample had an insufficient amount of extracted DNA whilst the median yield was 3.6 µg (range 0.075–59.2). Overall, real-time Ion Torrent sequencing results were obtained for 26 (74%) of the 35 patients (Fig. 1b). The median global turnaround time (TAT) from patient biopsy to sequencing results was 51 working days (range 16–146) whilst the median delivery TAT from sample reception at the sequencing facility to final NGS report was 9 working days (range 5–17) (Fig. 1e). There were no significant differences in TAT between the recruiting centres (Fig. 1f and g).

**Table 1:** Clinical and pathological characteristics of patients and biopsies (*n* = 35)

Characteristics	Number of patients (%)
Age (years)	
Median	56
Range	23–73
ECOG performance status^a^	
0	16 (45)
1	17 (49)
2	2 (6)
Breast cancer subtype^a^	
ER+	20 (57)
HER2+	7 (20)
ER-/HER2-	8 (23)
Number of prior lines of therapy	
0	6 (17)
1	5 (14)
2	4 (11)
3	6 (17)
>3	14 (41)
Number of metastatic sites	
1	8 (23)
2	10 (28)
3	8 (23)
>3	9 (26)

^a^ ECOG; Eastern Cooperative Oncology Group, ER; oestrogen receptor evaluated by IHC, HER2; human epidermal growth factor receptor 2 evaluated by IHC and FISH. The breast cancer subtypes are based on the characteristics of the metastatic lesions

### Mutation detection from the Ion Torrent OncoDEEP clinical cancer panel and CNA using SNP arrays

**Fig. 2:**
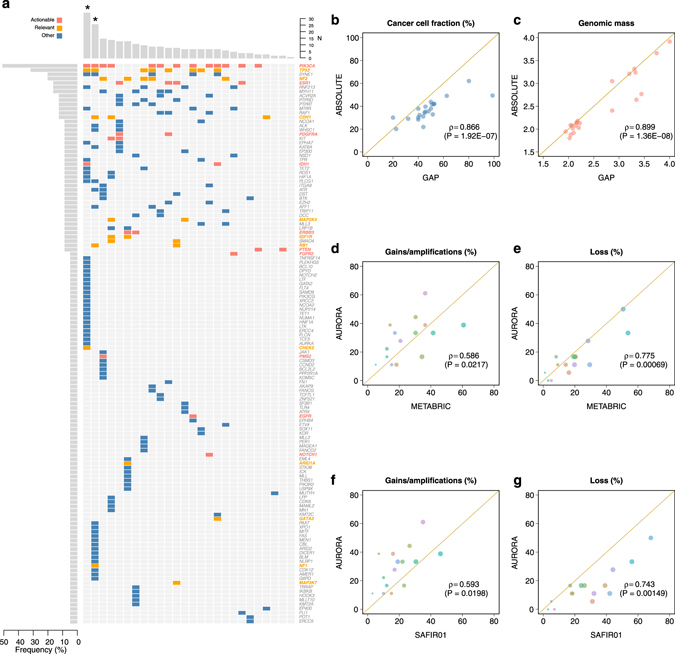
Mutation detection from the OncoDEEP clinical cancer panel and CNA from SNP arrays. **a** genes for which at least one mutation was indexed across the 26 patients, **b** and **c** comparison of cancer cell fraction and genomic mass respectively obtained from two widely used algorithms for estimating CNA from SNP arrays. In **a**, *asterisk* indicates potentially hypermutated patients. In **b** and **c**, each *dot* represents a sample. In **d**–**g**, each *dot* represents a gene from the list of clinically actionable or biologically relevant targets and is the mean of 100 bootstrap replicates such that for each replicate, the external cohorts are matched for ER and HER2 status. The size of each dot is proportional to the standard error of the mean frequency estimate

Quality control metrics for the sequencing data are provided in Supplementary Figure S1. The target regions were covered on average at 1758X (494–3075X) sequencing depth. Non-synonymous somatic mutations were called from the OncoDEEP clinical cancer panel in exons covered by at least 100X sequencing depth and a fixed threshold of 10% variant allele fraction (VAF). In total, 128 unique genes harboured at least one mutation representing one third of the panel (Fig. 2a). The median number of mutations indexed per patient was 6 (range 0–35) and the overall mutation detection rate i.e., percentage of patients where at least one mutation could be indexed, was 96%. Only 8 (4.15%) of the 193 mutations had previously been described in release v.76 of COSMIC in any type of cancer whilst 178 (92.23%) mutations had never been reported in the literature. The most frequently mutated genes were *PIK3CA* (50%), *TP53* (31%), *SYNE1* (19%), and *NF2* (15%).

We evaluated two established methods to call CNA from SNP arrays and obtained similar results (Fig. 2c and d) thereby attesting to the robustness of SNP arrays for the estimation of CNA. All the patients presented with at least one CNA and the CNA frequencies were comparable to those reported in the literature (Fig. 2d–g). For instance, 13 (72%) patients harboured a gain or an amplification of chromosome 8q where the *MYC* oncogene resides whilst 9 (50%) had a deletion of 17p where *TP53* is located.

Two patients presented with a higher than expected number of mutations. The integration of the sequencing and CNA data obtained from these two outlier patients is shown in Supplementary Figure S2. In general, the distributions of VAF and cancer cell fractions (CCF) were high and tightly clustered, indicative of genuine somatic mutations. For the whole cohort, *ERBB2* amplification status was assessed centrally using FISH whilst *PTEN* loss was evaluated by immunohistochemistry (IHC). These single assay data were compared with CNA obtained from the SNP arrays and the results are shown in Supplementary Figures S3 and S4. No false negative calls were recorded for *ERBB2* amplification status comparing SNP arrays to FISH data. However, 7 of 18 patients (39%) effectively harboured supernumerary copies of *ERBB2* (*n* ≤ 4 copies) which were missed by FISH assays owing to centromere 17 co-amplification or low level 17q polysomy. There was no concrete evidence of a correspondence between *PTEN* IHC staining results and the corresponding copy number level as determined by SNP array.

### Actionable alterations from targeted NGS and SNP arrays

Supplementary Tables S1 and S2 provide the definitions of actionable and biologically relevant alterations. 19 of 26 (73%) patients harboured at least one actionable mutation, such as PIK3CAp.H1047R or ESR1p.Y537N, whilst 8 (31%) had only one such mutation and six (23%) had at least one biologically relevant mutation and none that were clinically actionable (Supplementary Figure S5). Comparatively, 16 of 18 (89%) patients for whom the SNP arrays were available harboured at least one actionable CNA, such as *EGFR* amplification,^17, 18^ whilst 2 (11%) had only one such aberration and an equal number had at least one biologically relevant CNA and none that were clinically actionable. Using the combined information from the SNP arrays and the targeted sequencing, and focusing on the subset of strictly actionable alterations, 23 of 26 (88%) patients had at least one clinically actionable alteration whilst only 3 (12%) had only one such alteration. Overall, of the initial 7 patients without actionable mutations, four were found to harbour at least one actionable CNA.

### Orthogonal cross-testing of substitution calls using Illumina NGS

**Fig. 3:**
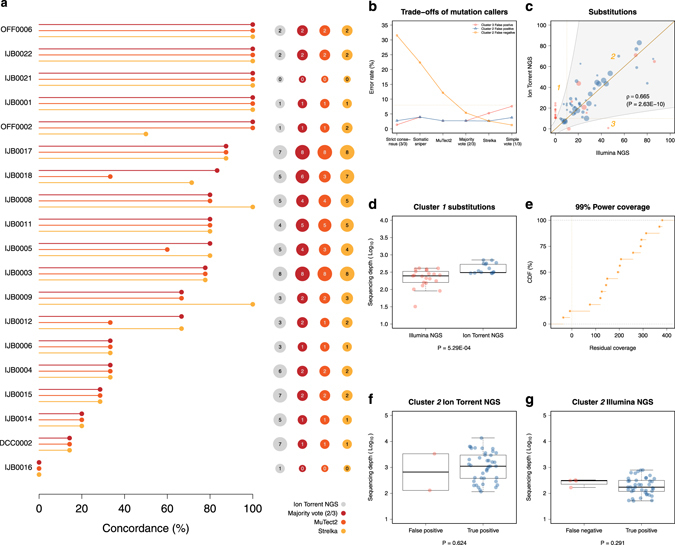
Orthogonal cross-testing of substitution calls using Illumina NGS. **a** distribution of % concordance for single nucleotide substitutions indexed from the Ion Torrent and Illumina NGS platforms using different mutation callers, **b** % error rate of the different mutation callers over three substitution clusters compared to the Ion Torrent sequencing, **c** correlation of VAF for substitutions indexed from the Ion Torrent and Illumina NGS using a majority rule of any two of three mutation callers, **d** comparison of sequencing coverage between the Ion Torrent and Illumina NGS for substitutions in cluster *1*, **e** empirical cumulative distribution of theoretical residual coverage from the Illumina sequencing for substitutions in cluster *1* and cluster *2* false negatives, **f** comparison of sequencing coverage from the Ion Torrent NGS for substitutions in cluster *2* false positives, and **g** comparison of sequencing coverage from the Illumina NGS for substitutions in cluster *2* false negatives. In **a**, the different mutation callers and any combination thereof are colour coded and indicated at the bottom. The leftmost panel gives the number of substitutions called. In **b**–**g**, the cluster numbers are relative to (**c**). Substitutions in clusters *1* and *3* are exclusive to one of the sequencing platforms and have 0% VAF in the alternate data. Substitutions in cluster *2* are those found by either or both NGS platforms and have non-zero VAF in both sequencing data. False negatives in cluster *2* are substitutions indexed by Ion Torrent NGS only whilst false positives are substitutions indexed by Illumina NGS only using a given mutation caller or any combination thereof. The size of each dots is proportional to the difference in coverage between the two sequencing platforms. In **e**, the residual coverage is obtained by subtracting the theoretical coverage required to achieve 99% power for indexing a substitution given one mutated copy out of *n* total copies from the observed value of sequencing depth. Only two substitutions in cluster *1* failed the criteria of positive residual for detection and are non-callable loci

The substitutions obtained from the Ion Torrent sequencing were compared to data generated from the Illumina NGS platform. The same DNA samples were sequenced and substitutions were called in overlapping regions using similar filtering criteria and a combination of three established mutation callers. Patient matched data were available for 19 (73%) patients and all three mutation callers were mostly consistent (Fig. 3a). All but one actionable substitution, NOTCH1p.D2082E in cluster *1* (see below), were concordant.

The substitutions were categorised into three clusters and the error rates of each mutation caller and several combinations thereof were benchmarked using this framework. Substitutions in clusters *1* and *3* are exclusive to one of the sequencing platforms and have 0% VAF in the alternate data, whereas substitutions in cluster *2* are those found by either or both NGS platforms and have non-zero VAF in both sequencing data. The definite calls were made for each substitution based on a majority vote of any two of the three mutation callers (Fig. 3b and c). This choice was guided by a low global Illumina specific false negative error rate on cluster *2* substitutions whilst maintaining a relatively low global Illumina specific false positive error rate on cluster *2* and *3* substitutions. Using this approach, a global concordance rate of 40.9% was achieved.

Substitutions in cluster *1* cannot be reconciled. There was a statistically significant difference in coverage between the Ion Torrent and Illumina NGS data for these substitutions (Fig. 3d). However, all but one of the substitutions were covered in excess of 100X in the Illumina NGS data and the residual coverage, which is the difference between the observed sequencing depth and the expected value at 99% statistical power to detect a mutation for given CCF and copy numbers, were positive for all except two substitutions (Fig. 3e). Similarly, two substitutions were called in cluster *3* which constitute Illumina specific false positives and were covered at 239X and 1668X using the Ion Torrent NGS platform. In total, only 2.7% of the substitutions in cluster *2* were Illumina specific false positives using the Ion Torrent platform as standard whilst 5.41% were Illumina specific false negatives. There were no statistically significant differences in sequencing coverage between these groups of substitutions and the true positives called by both platforms (Fig. 3f and g). Furthermore, similar to cluster *1* substitutions, all the Illumina specific false negatives had positive residual coverage (Fig. 3e). For comparison, the sequencing coverage and the percentage of non-reference bases at these particular loci in the normal matched samples are shown in Supplementary Figure S6.

### Comparison of CNA from targeted NGS and SNP arrays

**Fig. 4:**
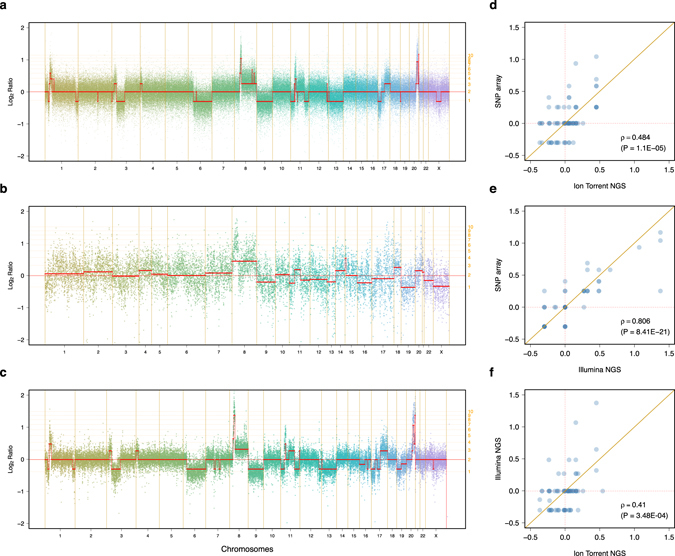
Genome-wide copy number profiles inferred from SNP arrays and targeted NGS data. Genome-wide Log_*2*_ ratio profiles of patient IJB0021 obtained using **a** SNP array **b** Ion Torrent and **c** Illumina targeted NGS data. The *solid vertical lines* represent chromosome boundaries whilst the *dashed horizontal lines* indicate canonical copy numbers inferred from the cancer cell fraction and ploidy of the sample, both estimated from the SNP array. The *solid horizontal lines* represent the segmented Log_*2*_ ratios. Correlation of segmented Log_*2*_ ratios between **d** the SNP array and the Ion Torrent platform, **e** the SNP arrays and the Illumina platform, and **f** the two NGS platforms for the same case patient. In **a**–**c**, the loci are sorted according to their coordinate on the human genome reference hg19/GRCh37. For ease of representation, they are plotted by indices

**Fig. 5:**
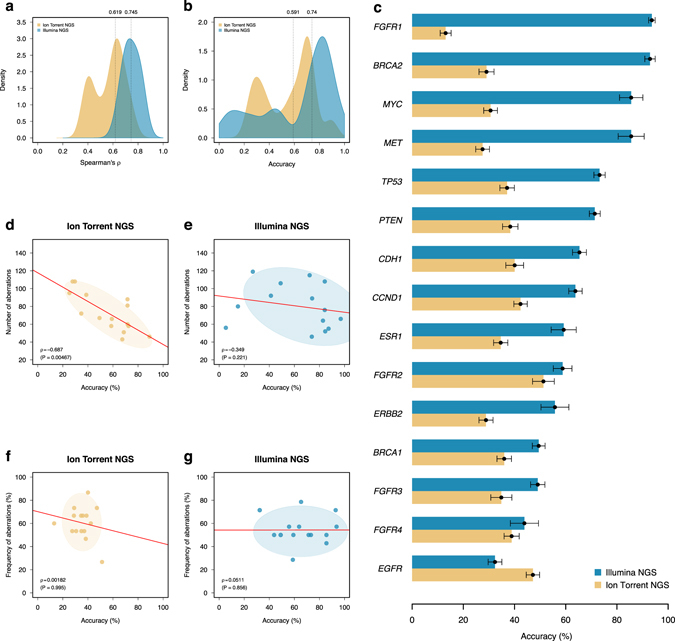
Comparison of copy number profiles from SNP arrays and targeted sequencing data. **a** distribution of Spearman’s correlation coefficient comparing the segmented Log_*2*_ ratios from the SNP arrays and the NGS data across patients with all three data types. The segmented Log_*2*_ ratios were further categorised into copy number aberration calls i.e., −1, 0 and +1 and compared patient wise between the SNP arrays and the NGS data. The resulting distributions of accuracy values are depicted in **b**. For a small set of 15 clinically actionable or biologically relevant genes, the accuracy values comparing SNP arrays and NGS data were computed using all available samples and displayed individually for each gene in **c**. **d**, **e** correlation of accuracy values measured genome-wide for each patient as in **b** vs. the number of aberrations as determined by the corresponding NGS platform. **f**, **g** correlation of accuracy for each of the 15 clinically actionable or biologically relevant genes vs. the frequency of aberrations for the same genes measured using the SNP array. In **c**, each value of accuracy was generated by 100 bootstrap replicates. The values displayed represent the mean of the replicates and the error *bars* represent the standard error of this estimate

The CNA obtained from the Ion Torrent sequencing were also compared to the profiles obtained from SNP arrays and Illumina NGS. Fig. 4a–c show the Log_*2*_ ratios from the SNP array and both NGS platforms for a case patient. The segmented Log_*2*_ ratios from the SNP array and Illumina sequencing clustered, as expected, into canonical copy number genotypes whilst the segmented Log_*2*_ ratios obtained from the Ion Torrent NGS platform were spread between those canonical genotypes leading to relatively poor correlation in Log_*2*_ ratio data space (Fig. 4d and e). Nonetheless, this approach was applied to each copy number platform for 14 (54%) patients for whom all three data types were available and the segmented Log_*2*_ ratios were compared genome-wide using the Spearman’s correlation. The distribution of correlation coefficients comparing the SNP array to the NGS data was bimodal for the Ion Torrent platform with 5 (29%) samples displaying a poor correlation of *ρ* < 0.5 (Fig. 5a). The median correlation coefficients were *ρ* = 0.615 and *ρ* = 0.745 for the Ion Torrent and Illumina NGS platforms respectively.

In order to further evaluate the ability of both NGS platforms to call CNA, the segmented Log_*2*_ ratios were grouped into three categories i.e. deletion (−1), copy neutral (0), gain/amplification (1) and further compared using the accuracy which is the sum of concordant calls relative to the total number of aberrations. Fig. 5b shows the distribution of accuracy values evaluated genome-wide for each patient. The average accuracy of CNA call was 59.1% for the Ion Torrent and 74.0% for the Illumina NGS platforms. Both distributions were bimodal with 6 (35%) and 5 (29%) patients showing low values of *α* < 0.5 and *α* < 0.6 for the Ion Torrent and Illumina NGS platforms, respectively. Lastly, the ability of both sequencing platforms to call CNA in particular genes of interest was evaluated on 15 clinically actionable or biologically relevant genes. For each gene, the CNA calls were pooled and the cohort-wise concordance measured as the accuracy of concordant calls. Fig. 5c contrasts the distribution of accuracy values observed across these 15 genes for both NGS platforms. We observed relatively high concordance rates using the Illumina NGS platform for genes frequently aberrant in breast cancers e.g., 90.1% (95% CI: 86.8–93.3) for *MYC*, 63.8% (95% CI: 61.4–66.2) for *CCND1* and 57.4% (95% CI: 52.3–62.5) for *ERBB2* whilst the corresponding values for the same genes were 29.5% (95% CI: 27.5–31.8), 40.2% (95% CI: 37.7–42.7) and 28.3% (95% CI: 25.6–30.9), respectively, using the Ion Torrent sequencing platform. There was a significant negative correlation between the accuracy and the number of aberrations per patient for the Ion Torrent data (Fig. 5d) but not for the Illumina NGS platform (Fig. 5e). There were no associations between the accuracy measured cohort-wise and the frequency of aberrations affecting the 15 genes of interest for either of the two NGS platforms (Fig. 5f and g).

## Discussion

The growing number of targeted anticancer therapies either approved or under clinical development has led to a wide interest in personalised treatment approaches in clinical practice.^19, 20^ However, many of these cancer molecular screening initiatives have had limitations. For instance, several of these screening programs used different sequencing techniques and very often lacked orthogonal validation. Together with varying concepts of what constitutes an actionable mutation, this makes the results difficult to compare. The current study is a pilot phase undertaken to evaluate the feasibility of AURORA, a pan-European molecular screening programme for advanced breast cancer patients.

The success rate for the primary endpoint of 63% compares favourably with existing literature.^5–7, 21^ The median global TAT of 51 working days was due to unforeseen delays either between patient consent and surgical biopsy or between collection of biological samples and shipment to the central pathology laboratory. In part, due to the above findings, these parameters are now rigorously monitored in the parent programme where the global TAT does not exceed 20 working days (personal communication). 73% of the patients harboured one or more actionable mutations and this increased to 88% when considering the underlying CNA. These numbers are encouraging on account of the availability of alterations that can be theoretically targeted despite limited clinical trial based evidence of benefit to patient survival from targeted therapy in breast cancer. Yet, the decision to prioritise a particular molecular target for trial allocation given several such alterations within the same patient constitutes a considerable challenge for which specific algorithms and combined expertise will be required in the future.

Regarding the secondary endpoint and considering clinically actionable mutations, our results are reassuring. All but one substitution was positive using both the Ion Torrent and Illumina sequencing platforms. Furthermore, from a pragmatic viewpoint, 5 (26%) sample pairs showed complete agreement. However, no single mutation caller was able to reproduce globally with 100% concordance the results obtained in real-time from the Ion Torrent NGS platform. The Illumina specific false negative and false positive rates over somatic substitutions with non-zero VAF in both data types can be reasonably contained whilst substitutions private to either sequencing technology despite adequate coverage in both data types were more frequent in the Ion Torrent data. Thus, from a technical perspective, our results are somewhat sobering and whilst the higher background error rate of the Ion Torrent NGS technology could be a contributing factor, the small number of samples and mutations assessed here do not allow for a full exploration of the possible causes of these discrepancies. Massively parallel sequencing using targeted gene panels has emerged as a technology that can be applied within a clinically meaningful TAT to identify known biomarkers of resistance or sensitivity to a given drug. In the broader context of AURORA, all the results are reported to investigators unaltered. However, our analysis shows the major limitations of this approach outside known mutation hotspots and in anticipation, the AURORA study design allows biobanking of frozen and residual FFPE material for later research purposes using alternative high-throughput technologies such as whole genome or exome sequencing.

To the best of our knowledge, no other studies have compared prospectively CNA obtained from targeted sequencing data to SNP arrays in a clinical setting. Our results show that the gold standard FISH assay for *ERBB2* is fully concordant with SNP arrays. However, the Illumina NGS platform outperforms the Ion Torrent technology when compared to the same SNP arrays. This is not unexpected since the Illumina NGS panel accommodates intergenic targets which, combined with the fact that hybrid capture methods for sequencing library preparation often carry over off-target reads, increases the effective number of regions where copy numbers can be evaluated. Several algorithms^22, 23^ take advantage of this, benefiting downstream CNA calling and translating into higher concordance with the denser SNP arrays. The combination of assays, such as mutation profiling and assessment of CNA, into one cost effective package is an attractive idea. Together with a low requirement of input DNA, as is often obtainable from clinical samples, makes for a compelling argument in favour of the Ion Torrent NGS platform. However, given the poor agreement with solid benchmarks such as SNP arrays, the results presented here do not support, at least for now, the targeted Ion Torrent NGS platform as a truly multipurpose assay in a clinical setting. Within AURORA, central pathology results reported to investigators in real-time include *ERBB2* IHC as well as FISH when applicable. This is clinically meaningful since *ERBB2* gene amplification remains, to date, the sole approved CNA biomarker in breast cancer. Sub-studies undertaken in the framework of the main AURORA programme include real-time SNP arrays and retrospective whole genome sequencing to alleviate the technical limitations of identifying CNA using the Ion Torrent technology and will allow the study of the clonal evolution of breast cancer.

Overall, our study contrasts the benefits and pitfalls of personalised molecular screening for patients with advanced cancer involving the combination of multiple high-throughput genomic techniques. In view of these results, greater effort is being devoted to improving the concordance of mutation calls between different NGS platforms and to harmonising CNA calls with SNP arrays in the ongoing AURORA programme. With recent findings on the clinical utility of circulating tumour DNA^11, 24^ and its implementation into the AURORA study design, it becomes imperative to thoroughly evaluate the technical feasibility of profiling circulating biomarkers to support clinical decision making.

## Methods

### Patients and samples

Patients were enroled in four European centres (Institut Jules Bordet, Belgium; Hospital Vall d’Hebron, Spain; Sana Klinikum Offenbach, Germany; and Dundee Cancer Centre, United Kingdom). The study was approved by the respective ethical committees from the named institutions and was performed in accordance with relevant guidelines and procedures. Patients considered eligible for this study were those (1) with histologically proven distant metastatic or locally recurrent invasive breast cancer (2) with an Eastern Cooperative Oncology Group Performance Status equal to or less than two, (3) with clinical and laboratory parameters safe for tumour biopsy, (4) for whom FFPE tumour tissue from a locally recurrent or metastatic lesion and whole blood for research purposes were available. The samples were centralised in real time at the European Institute of Oncology (Milan, Italy) where H&E slides were reviewed by a board-certified pathologist for the evaluation of cellularity. At the central laboratory, IHC for ER, PR, HER2, Ki67 and PTEN were performed using the ER pharmDx kit (Dako), the HercepTest kit (Dako), the mouse mAb anti h-Ki-67, the clone MIB-1 (Dako) and the rabbit mAb anti h-PTEN, clone 138G6 (Cell Signalling) respectively. FISH for *ERBB2* was performed using the HER2 FISH pharmDx kit (Dako) and scored according to the ASCO/CAP guidelines. Wherever applicable, macrodissections to enrich for tumour cells were performed. DNA was extracted from the tissue and blood samples using either the Qiagen DNA FFPE tissue kit and the QIAamp DNeasy blood and tissue kit, respectively, following the manufacturer’s instructions. The cut-off values for tumour content and DNA quantity were 10% and 400 ng, respectively. DNA concentrations were measured using the Qubit fluorometer (Life Technologies) following which, aliquots were shipped to (1) OncoDNA (Gosselies, Belgium) for sequencing using Ion Torrent NGS technology, (2) the Wellcome Trust Sanger Institute (Hinxton, UK) for sequencing using Illumina NGS technology, and (3) the J.-C. Heuson Breast Cancer Translational Research Laboratory (Brussels, Belgium) where the Affymetrix OncoScan FFPE Express arrays were performed. The Illumina NGS and Affymetrix SNP arrays were performed as batch processes whilst the sequencing and delivery of results from OncoDNA were monitored in real time.

### Targeted gene screen using Ion Torrent NGS

Somatic mutations were assessed using the OncoDEEP clinical cancer panel which is a validated AmpliSeq design panel targeting the exonic regions of 409 cancer related genes (Supplementary Table S3). The same protocol was applied to DNA extracted from FFPE tumour and whole blood normal matched samples. Briefly, the targeted sequencing libraries were generated using the Ion AmpliSeq library kit 2.0 according to the manufacturer’s instructions (Life Technologies) using 80 ng of genomic DNA. The primers used for amplification were partially digested by *Pfu* restriction enzyme and the digestion products were ligated to barcoded adaptors and purified using Ampure Beads. The purified products were amplified for five cycles and purified once more using Ampure Beads. The quality of the libraries was assessed using a qPCR following which 10 pM of each library underwent emulsion PCR using an IonChef system. The chips were loaded on an Ion PGM and were sequenced at a target coverage of 500X.

### Targeted gene screen using Illumina NGS

The exonic regions of 371 cancer related genes (Supplementary Table S4) were enriched using a custom design of SureSelect RNA baits following the manufacturer’s instructions (Agilent). The same protocol was applied to DNA extracted from tumour and normal matched samples. Briefly, 500 ng of genomic DNA was fragmented to an average insert size of 145 bp (75–300 bp) and subjected to Illumina DNA sequencing library preparation using the Bravo automated liquid handling platform. Individual samples were indexed using a unique DNA barcode via six cycles of PCR. Equimolar pools of 16 libraries were prepared and hybridised to the custom RNA baits and sequenced using an Illumina HiSeq device in 75 bp paired-end mode at a target coverage of 200X.

### Copy number aberration profiling using SNP arrays

Copy number aberration profiling using the SNP arrays was performed according to the manufacturer’s instructions (Affymetrix). In short, the molecular inversion probes (MIP) were incubated with the FFPE extracted DNA at 58°C overnight after an initial denaturation at 95°C for 5 min. Each sample was then split into two aliquots and a gap fill reaction was performed. Uncircularised MIP and genomic DNA were digested using a cocktail of exonucleases. The remaining circular MIP were then linearised using a cleavage enzyme and amplified by PCR. Following a second round of PCR amplification, the 120 bp amplicons were cleaved into two fragments with the *Hae*III enzyme. The samples were then mixed with the hybridisation buffer and injected into the arrays where they were allowed to hybridise at 49°C for 16–18 h. At the end of the hybridisation period, the arrays were stained and washed using the GeneChip Fluidics Station 450 and loaded into the GeneChip Scanner 3000 where array fluorescence intensity was scanned to generate binary CEL files using the Affymetrix GeneChip Command Console.

### Bioinformatics analyses

#### Mutation calling from Ion Torrent targeted gene screen

Sequence reads from the tumour and matched normal samples were aligned against the human genome reference version hg19/GRCh37 using the Ion Torrent TMAP aligner with default parameter settings. Mutations were called from the resulting BAM files using the Torrent Suite variant caller (Life Technologies) with the default settings of the ‘Somatic High Stringency’ pipeline and cross-checked using the NextGENe software (Softgenetics) using the ‘Ion Torrent’ predefined pipeline. Germline mutations were filtered by subtracting variants found in the matched normal sample from those called in the corresponding tumour sample. The resulting somatic mutation calls were further filtered to exclude variants (1) that were not sequenced in both sense with a minimum ratio of 10/90%, (2) with less than 100 read depth, and (3) variant allele fractions lower than 10% in the tumour sample. For an alpha list of target genes (Supplementary Table S5), mutations occurring below 10% VAF were accepted. Manual evaluation was done for indels using IGV.^25^

#### Mutation calling from Illumina targeted gene screen

Sequence reads from the tumour and matched normal samples were pre-processed according to the GATK best practices. The raw reads were aligned against the human genome reference hg19/GRCh37 using the BWA aligner.^26^ Duplicate reads were marked using Picard following which the data were filtered to keep only properly paired and mapped reads with mapping quality greater than 60. Sequence reads around potential indels were locally realigned and the base quality scores were recalibrated using GATK.^27^ Mutations were called from the resulting BAM files using MuTect2,^28^ SomaticSniper^29^ and Strelka^30^ in matched tumour/normal mode using the default parameter settings for each of the aforementioned algorithms. The resulting somatic mutation calls were further filtered to include only variants (1) occurring in regions common to both Ion Torrent and Illumina sequencing panels, (2) having a VAF above 10% in the tumour sample, and (3) having a read depth greater than 50X. Similar to the Ion Torrent NGS, mutations occurring in the alpha list (Supplementary Table S5) were accepted if they occurred with a VAF below 10%. The resulting lists of mutation calls were considered individually for each mutation caller or combined by voting using (1) a simple rule of a mutation indexed by at least one of the three, (2) a majority rule of a mutation called by at least two of the three, and (3) a strict consensus of all three mutation callers. In downstream comparison, we considered only substitutions on account of the known issues of calling indels with confidence from Ion Torrent NGS data.

#### Copy number aberration analysis using SNP arrays

The raw intensity values were normalised to obtained Log_*2*_ ratios, B Allele Frequencies (BAF) and genotyping calls (AA/AB/BB) using Affymetrix Power Tools. We used release NA.33 of the NetAffx library for the reference model and annotation. We computed the Median Absolute Pairwise Deviation and the Median Auto-Correlation from the Log_*2*_ ratios as quality control metrics and used a threshold of 0.40 and 0.45, respectively, to discard failed arrays. We used two parallel approaches involving (a) allele specific copy number analysis using heterozygous SNP probes and (b) total copy number analysis using the full set of 200 K markers and parameters from (a) to control for the cancer cell fraction and genomic mass. From the BAF and genotyping calls, only informative SNP probes displaying heterozygous genotype (AB) and 0.1 < BAF < 0.9 were kept for analysis at (a). The Log_*2*_ ratios and BAF were smoothed using the median absolute deviation and segmented jointly using a multitrack segmentation algorithm from the library copynumber^31^ to determine common breakpoints. Estimates for the cancer cell fraction and genomic mass were obtained using GAP^32^ and compared to the results obtained from ABSOLUTE^33^. Samples with a cancer cell fraction lower than 30% were further excluded. For analysis at (b), the Log_*2*_ ratios for the same samples analysed at (a) were segmented by penalised least square regression as above and non-rounded estimates of copy numbers were obtained as

$$y = \frac{1}{\alpha }\left( {{2^{\frac{x}{c}}}\left( {{\psi}\alpha \,{\rm{ + }}\,{\rm{2}}\left( {1 - \alpha } \right)} \right) - 2\left( {1 - \alpha } \right)} \right)$$

where *α* is the CCF and *ψ* is the genomic mass, both estimated at (a). *c* = 0.8 is a constant representing the compression ratio of the array and finally *x* is the observed Log_*2*_ ratio of a given segment. The copy numbers were categorised as deletions (−1) if *y* < *ψ*−0.5, gains (+1) if *y* > *ψ* + 0.5, amplifications (+2) if *y* > *ψ* + 2.5, and copy neutral (0) otherwise. Unless otherwise stated, all parameter settings were kept at default values and all computations were done using R/Bioconductor.

#### Copy number aberration analysis using targeted NGS

The read counts from aligned and sorted BAM files of the Ion Torrent sequencing were processed using ONCOCNV^34^ with default parameter settings to correct for library size, GC content and amplicon length. The pool of normal samples was used as baseline control to capture the technology specific bias against which each tumour sample was normalised to obtain an estimate of Log_*2*_ ratio. The Log_*2*_ ratios were segmented using the circular binary segmentation^35^ and CNA calls were obtained using the default clustering approached implemented in ONCOCNV. The read counts from the aligned and sorted BAM files of the Illumina sequencing were processed using cnvkit^22^ to obtain an estimate of Log_*2*_ ratio for on and off target regions. The pool of normal samples was used as baseline control against which each tumour sample was normalised. The Log_*2*_ ratios were smoothed by median absolute deviation and segmented by penalised least square regression using the library copynumber.^31^ Estimates of cancer cell fraction and genomic mass were obtained using ABSOLUTE^33^ and non-rounded estimates of copy numbers and copy number calls were obtained as described above for SNP arrays. Unless otherwise stated, all parameter settings were kept at default values.

### Statistical analyses

Integration of the VAF of mutations indexed by the Ion Torrent NGS platform and CNA profiles from the matched SNP arrays to compute the Log-Likelihood and individual CCF of each mutation was done using ABSOLUTE.^33^ Similarly, the sequencing depth required to achieved 99% statistical power to call a given base was computed using ABSOLUTE^33^ with an error rate *E* = 2*E*-03 and an FDR = 5E-07. The frequencies of aberration calls and the percentage accuracy were obtained by bootstrapping. The values displayed are mean estimates and confidence intervals are standard errors. The concordance of mutation calls for substitutions was evaluated using the simple matching coefficient. All correlations were measured using the non-parametric Spearman’s ρ coefficient and all statistical hypothesis tests were done using the non-parametric Wilcoxon’s rank sum test. *P*-values were two sided and paired or unpaired as appropriate. Unless otherwise stated, all computations were done in R/Bionconductor.

### Data availability

The sequencing and SNP arrays data have been deposited at the European Genome-Phenome Archive (http://www.ebi.ac.uk/ega/) which is hosted by the European Bioinformatics Institute, under accession number EGAD00001000870.

## Electronic supplementary material

Supplementary Figures and Tables Legends

Supplementary Figure S1

Supplementary Figure S2

Supplementary Figure S3

Supplementary Figure S4

Supplementary Figure S5

Supplementary Figure S6

Supplementary Table S1

Supplementary Table S2

Supplementary Table S3

Supplementary Table S4

Supplementary Table S5
