# Supplementary material for: The AURORA pilot study for molecular screening of patients with advanced breast cancer–a study of the breast international group
Source: NPJ Breast Cancer. 2017 Jun 29;3:23. doi: 10.1038/s41523-017-0026-6 (PMC5491498; doi:10.1038/s41523-017-0026-6)
Supplement: Supplementary file 1 — Supplementary Figures and Tables Legends [file 41523_2017_26_MOESM1_ESM.docx]

# Supplementary Figures

**Supplementary Figure S1: Distribution of sequencing coverage.** (a) cumulative distribution of sequencing coverage for the tumour samples using the Ion Torrent NGS platform, (b) density plot for the 25%, 50% and 75% percentiles of sequencing coverage depth across all samples, (c) same as (a) for the normal matched samples, (d) same as (b) for the normal matched samples, and (e) to (h) same as (a) to (d) respectively for the Illumina sequencing platform. The empirical CDF measures the cumulative percentage of targets covered by at least the sequencing depth specified on the x axis. Each curve represents a sample. Moving rightwards, the percentage of targets with a given coverage increases. The sequencing by Illumina targeted NGS was carried out in two batches at different target coverages. This causes the separation of the curves in (g) and contributes to the bimodal distributions observed in (f) and (h).

**Supplementary Figure S2: Integration of copy numbers and mutations to compute the cancer cell fraction in two potentially hypermutated patients.** (a) maximization of the log-likelihood of observed VAF for all mutations indexed over a range of possible cancer cell fractions for patient IJB0019, (b) scatter plot of VAF against cancer cell fraction for each mutation obtained by using the global cancer cell fraction for the same patient in (a), (c) distribution of VAF from patient IJB0002, and (d) frequency of mutations grouped by type of substitution for the same two case patients. In (b), the parental and absolute copy number status for the mutated loci were obtained from the corresponding SNP array and are represented as colour coded dilution curves. Although this does not represent an objective criterion, the fact that all the mutations had tightly clustered VAF and individual cancer cell fractions after integrating with CNA performed independently supposes that they are genuine mutation calls.

**Supplementary Figure S3: Comparison of *ERBB2* FISH and *PTEN* IHC with SNP arrays.** (a) comparison of CNA obtained using SNP arrays with *ERBB2* FISH (top) and *PTEN* IHC (bottom). The colour key is displayed in the bottom left corner. -1; loss, +1; low level gain, and +2; amplification. Grey represents negative results or no CNA. Cohort-wise frequency of CNA for chromosome (b) 10 and (c) 17, (d) *Log_2_* ratio against B allele frequency for patient IJB0008. In (d), each circle represents a genome segment, the size of which is proportional to the length of the segment. The segments belonging to chromosomes 10 and 17 are highlighted in orange whilst canonical copy numbers are displayed as dashed horizontal lines with the corresponding scale shown on the right. (e) and (f) *Log_2_* ratio profiles for chromosomes 10 and 17 respectively of patient IJB0008. In (b), (c), (e) and (f) the genomic coordinates of *PTEN* and *ERBB2* are marked by solid vertical lines whilst the coordinates are those of the human genome reference hg19/GRCh37. The centromeric region is indicated by a dotted vertical line. (e) and (f) are shown on the same vertical scale as (d). In the case of *ERBB2*, FISH identifies as positives only samples with high level copy number amplifications as determined by SNP array. Samples with simple low-level gains of 17q polysomy or amplification of the centromere are negatives by FISH. This is further exemplified in Supplementary Figure S4.

**Supplementary Figure S4: Case examples of *ERBB2* FISH negative and SNP array positive patients.** (a) and (c) *Log_2_* ratio versus B allele frequency for patient IJB0020 and VDH0003 respectively, (b) and (d) *Log_2_* ratios across chromosome 17 for the same patients. In (a) and (c), the segments belonging to chromosome 17 are coloured orange and the scale indicating canonical copy numbers is indicated on the right. In (b) and (d), the coordinates are those of the human reference genome hg19/GRCh37. The centromeric region is indicated by a dotted vertical line and the genomic coordinate of *ERBB2* is indicated by a solid vertical line. In (a) and (b), patient IJB0020 displays chromosome 17q polysomy and it is likely that the centromeric region is also amplified which would explain the negative FISH results. In (c) and (d), patient VDH0003 displays only a low-level copy number gain. In (a) and (b), patient IJB0020 shows a subclonal CNA with a *Log_2_* ratio of 0.47 for 17q. If $\alpha=\alpha_{3}+\alpha_{4}$ where $\alpha_{n}$ represents the % of total cells with $n$ copies of 17q and assuming $\psi\approx\psi_{3}\approx\psi_{4}$where $\psi_{n}$is the genomic mass of cells with $n$ copies of 17q, one can find the algebraic solution as $\alpha_{3}=0.29$ and $\alpha_{4}=0.36$ which represents a mixture of approximately 45% tumour cells with $n=3$ copies and 55% tumour cells $n=4$ copies.

**Supplementary Figure S5: Combined use of SNP arrays and targeted sequencing.** (a) sector plot of the % of patients with clinically actionable and biologically relevant mutations indexed from the Ion Torrent NGS platform, (b) same as (a) for CNA indexed from the SNP arrays, (c) combined sector plot of the % of patients with clinically actionable mutations or CNA, and (d) alteration map of CNA (top) and mutations (bottom) for clinically actionable and biologically relevant genes.

**Supplementary Figure S6: Reconciling false positive and false negative calls in cluster *2* substitutions.** (a) steps undertaken to automatically evaluate apparent false positive and false negative calls, (b) comparison of sequencing coverage for the Illumina specific false negatives of cluster *2* in the corresponding normal matched samples using the Illumina NGS data, (c) comparison of sequencing coverage for the Illumina specific false positives of cluster *2* in the corresponding normal matched samples using the Ion Torrent NGS data, (d) comparison of non-reference base fraction between the tumour and normal matched samples for cluster *2* positives using the Illumina NGS platform, (e) same as (d) using the Ion Torrent NGS data, (f) and (g) comparison of non-reference base fraction for cluster *1* to *3* discordant substitutions from the normal samples, (h) distribution of non-reference base fraction 5 bp upstream and downstream of each cluster *2* substitution. The bars represent mean estimates and are coloured according to the proportion of the 6 types of substitutions. The colour key is given in the top right corner. At each position, the left bar represents the tumour sample and the right bar represents the normal sample. The top row are error rates for the Illumina NGS platform and the bottom row represent error rates for the Ion Torrent sequencing platform.

# Supplementary Tables

**Supplementary Table S1: List of genes from the OncoDEEP clinical cancer panel considered as actionable**

**Supplementary Table S2: List of genes from the OncoDEEP clinical cancer panel considered as biologically relevant**

**Supplementary Table S3: List of 409 cancer related genes targeted by the Ion Torrent OncoDEEP clinical panel**

**Supplementary Table S4: List of 371 cancer related genes targeted by the Illumina custom panel**

**Supplementary Table S5: Alpha list of target genes for which mutations <10% VAF were accepted**
