# Supplementary figures and images for: The AURORA pilot study for molecular screening of patients with advanced breast cancer–a study of the breast international group
Source: NPJ Breast Cancer. 2017 Jun 29;3:23. doi: 10.1038/s41523-017-0026-6 (PMC5491498; doi:10.1038/s41523-017-0026-6)

**a**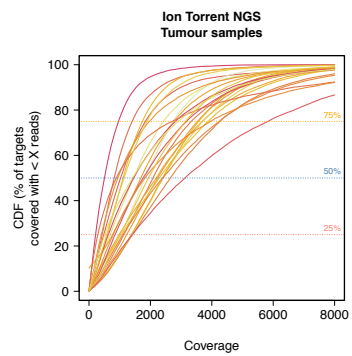**b**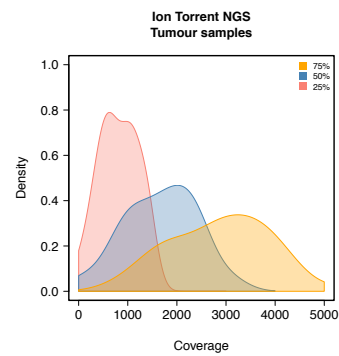**e**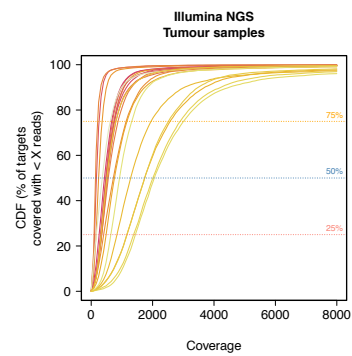**f**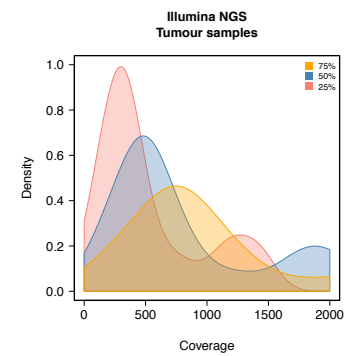**c**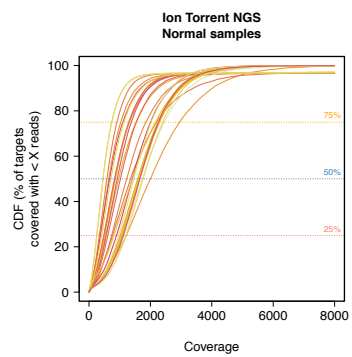**d**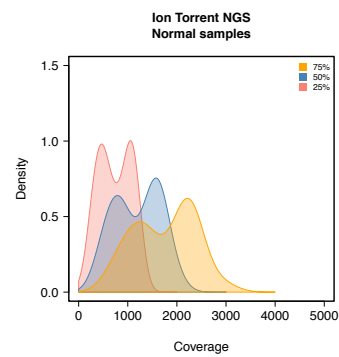**g**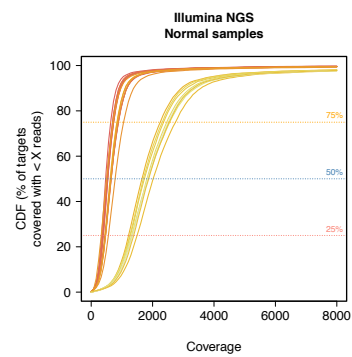**h**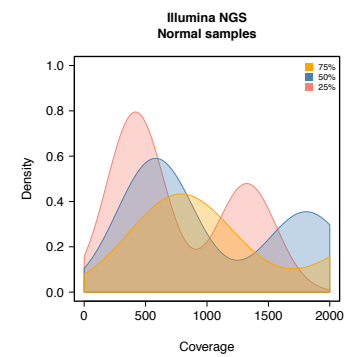

Supplement: Supplementary file 2 — Supplementary Figure S1 [file 41523_2017_26_MOESM2_ESM.pdf]

**a**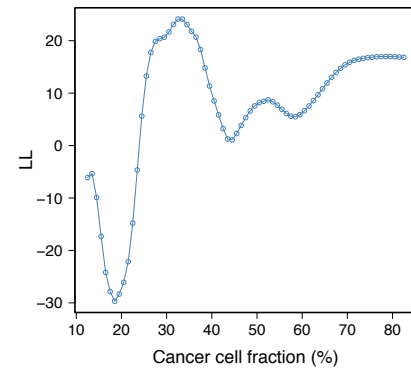**b**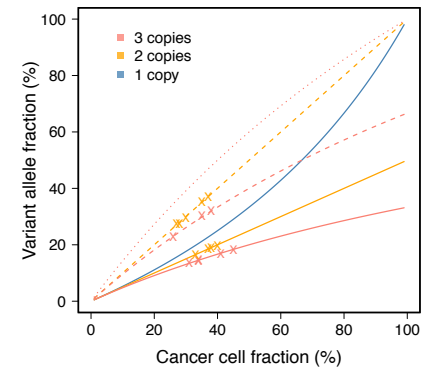**c**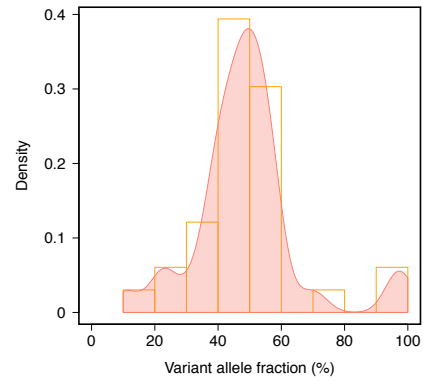**d**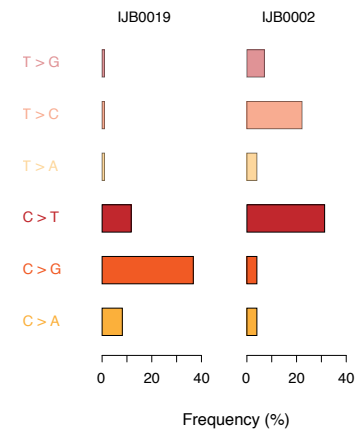

Supplement: Supplementary file 3 — Supplementary Figure S2 [file 41523_2017_26_MOESM3_ESM.pdf]

**a**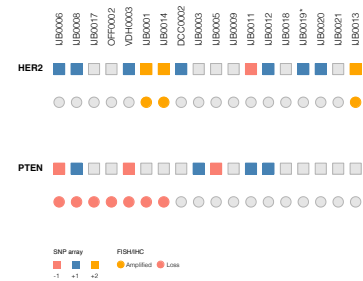**b**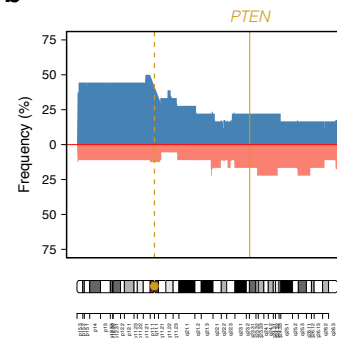**c**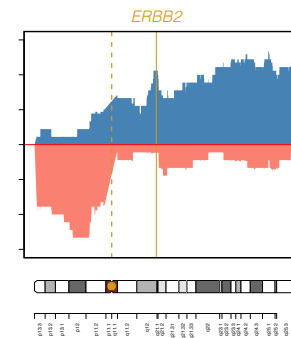**d**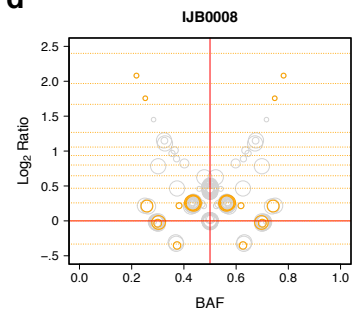**e**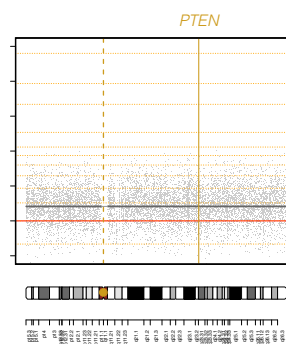**f**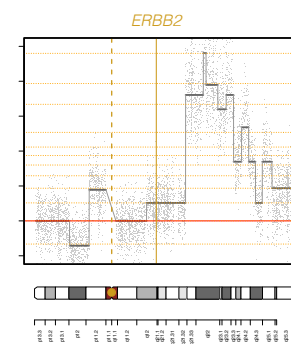

Supplement: Supplementary file 4 — Supplementary Figure S3 [file 41523_2017_26_MOESM4_ESM.pdf]

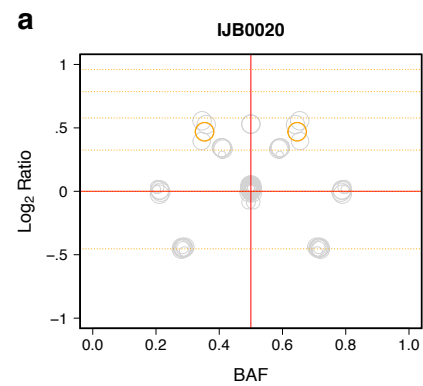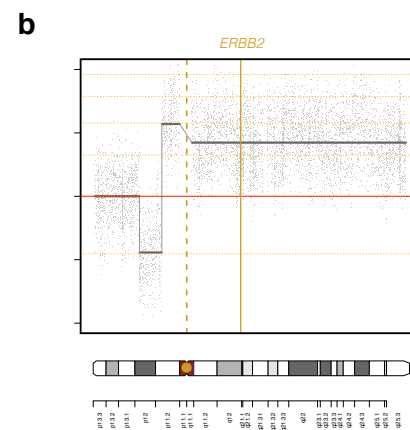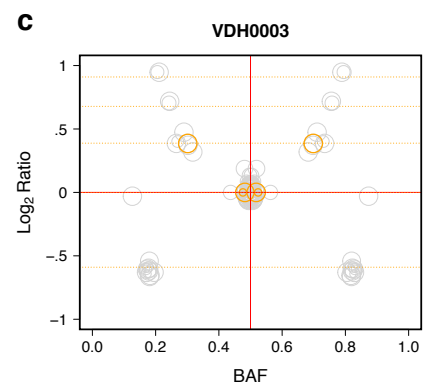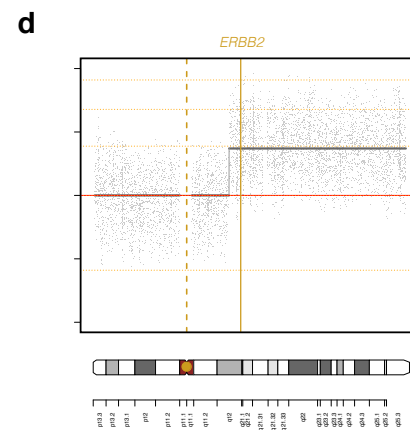

Supplement: Supplementary file 5 — Supplementary Figure S4 [file 41523_2017_26_MOESM5_ESM.pdf]

**a**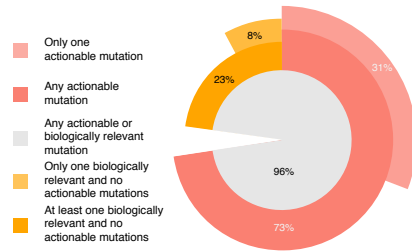**b**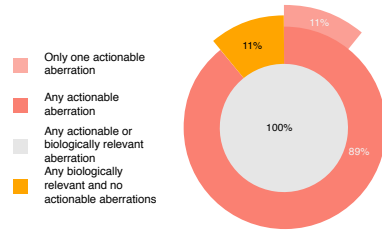**c**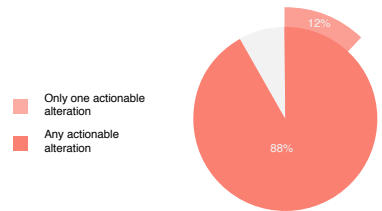**d**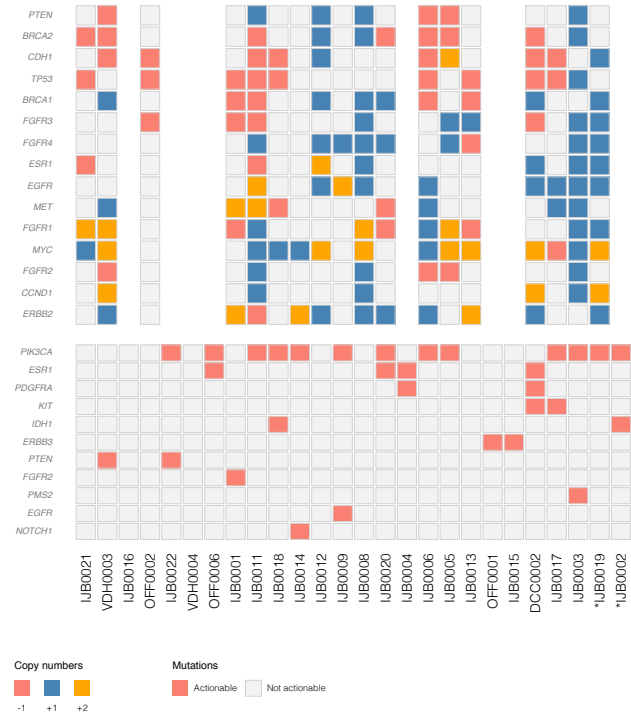

Supplement: Supplementary file 6 — Supplementary Figure S5 [file 41523_2017_26_MOESM6_ESM.pdf]

**a**

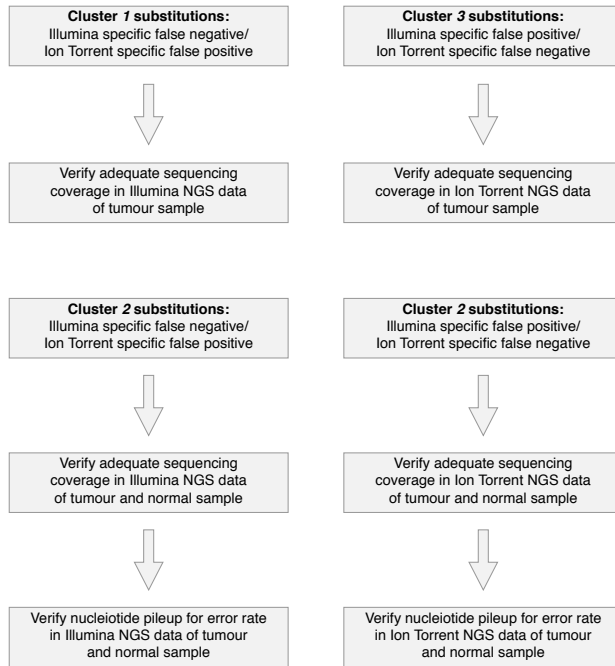

**b**

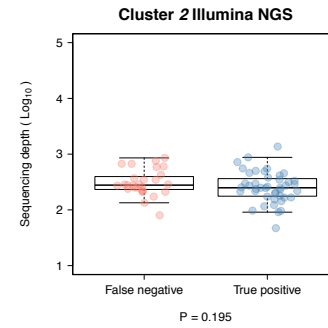

**c**

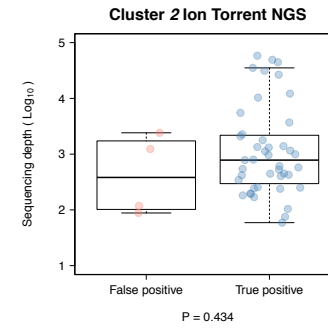

**d**

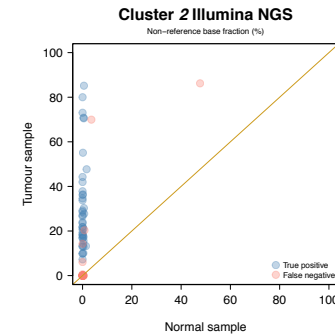

**e**

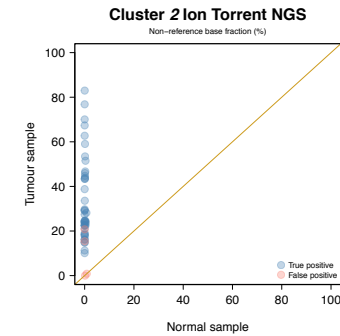

**h**

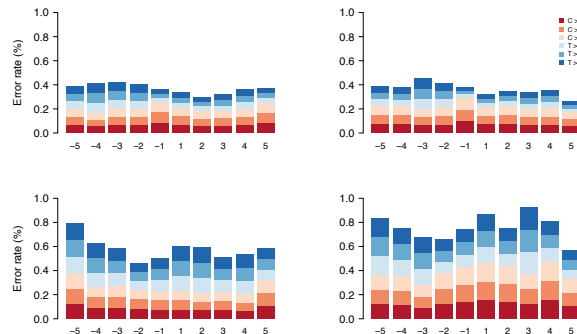

**g**

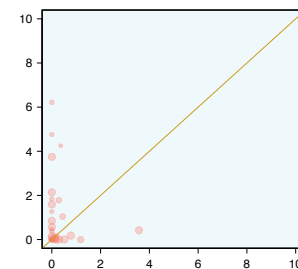

**f**

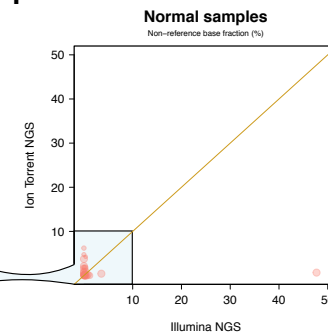

Supplement: Supplementary file 7 — Supplementary Figure S6 [file 41523_2017_26_MOESM7_ESM.pdf]
